# Chronic traumatic encephalopathy is a common co-morbidity, but less frequent primary dementia in former soccer and rugby players

**DOI:** 10.1007/s00401-019-02030-y

**Published:** 2019-06-01

**Authors:** Edward B. Lee, Kevin Kinch, Victoria E. Johnson, John Q. Trojanowski, Douglas H. Smith, William Stewart

**Affiliations:** 1 Translational Neuropathology Research Laboratory, University of Pennsylvania, Philadelphia, PA 19104 USA; 2 Department of Neuropathology, Queen Elizabeth University Hospital, 1345 Govan Rd, Glasgow, G51 4TF UK; 3 Department of Neurosurgery, Penn Center for Brain Injury and Repair, Perelman School of Medicine, University of Pennsylvania, Philadelphia, PA 19104 USA; 4 Department of Pathology and Laboratory Medicine, Center for Neurodegenerative Disease Research, Institute on Aging, Perelman School of Medicine, University of Pennsylvania, Philadelphia, USA; 5 Institute of Neuroscience and Psychology, University of Glasgow, Glasgow, G12 8QQ UK

**Keywords:** Chronic traumatic encephalopathy, Traumatic brain injury, Neurodegeneration, Dementia, Alzheimer’s disease, Tau

## Abstract

Chronic traumatic encephalopathy (CTE) is reported at high prevalence in selected autopsy case series of former contact sports athletes. Nevertheless, the contribution of CTE pathology to clinical presentation and its interaction with co-morbid neurodegenerative pathologies remain unclear. To address these issues, we performed comprehensive neuropathology assessments on the brains of former athletes with dementia and considered these findings together with detailed clinical histories to derive an integrated clinicopathological diagnosis for each case. Consecutive, autopsy-acquired brains from former soccer and rugby players with dementia were assessed for neurodegenerative pathologies using established and preliminary consensus protocols. Thereafter, next of kin interviews were conducted to obtain detailed accounts of the patient’s clinical presentation and course of disease to inform a final, integrated clinicopathological diagnosis. Neuropathologic change consistent with CTE (CTE-NC) was confirmed in five of seven former soccer and three of four former rugby players’ brains, invariably in combination with mixed, often multiple neurodegenerative pathologies. However, in just three cases was the integrated dementia diagnosis consistent with CTE, the remainder having alternate diagnoses, with the most frequent integrated diagnosis Alzheimer’s disease (AD) (four cases; one as mixed AD and vascular dementia). This consecutive autopsy series identifies neuropathologic change consistent with preliminary diagnostic criteria for CTE (CTE-NC) in a high proportion of former soccer and rugby players dying with dementia. However, in the majority, CTE-NC appears as a co-morbidity rather than the primary, dementia causing pathology. As such, we suggest that while CTE-NC might be common in former athletes with dementia, in many cases its clinical significance remains uncertain.

## Introduction

There is growing concern over the association between exposure to traumatic brain injury (TBI) and increased risk of a variety of neuropsychiatric and neurocognitive outcomes, in particular those linked to a specific neurodegenerative pathology known as chronic traumatic encephalopathy (CTE) [11, 29, 31, 42, 50]. First recognized in the early part of the twentieth century as the punch drunk syndrome of boxers [24], it was not until descriptions of CTE in autopsy studies of non-boxer athletes that the lifelong consequences of exposure to TBI attracted widespread attention [27, 36]. Nevertheless, despite this increased attention, there remain comparatively few accounts of the neuropathology of late survival from TBI [43]. As such, reflecting the relative paucity of cases described, consensus criteria for the neuropathological assessment of CTE remain preliminary [26] and the associated clinical consequences of this pathology remain unclear [48, 50].

In his report on the punch drunk syndrome, Harrison Martland provided the first, formal account of the chronic motor and neuropsychiatric consequences of exposure to repetitive mild TBI [24]. Thereafter, autopsy studies on single cases and small case series described the neuropathology of dementia pugilistica (DP) as an apparently distinctive, perivascular deposition of hyperphosphorylated tau, in the form of neurofibrillary tangles [3, 4, 11, 12]. In addition, widespread amyloid-β (Aβ) plaques were reported in a majority [11, 40]. However, although isolated studies in non-boxer individuals exposed to repetitive TBI were reported [6, 12], to the end of the twentieth century accounts of DP largely were restricted to observations in former boxers.

More recently, the pathology of DP, now termed CTE, has been documented in growing numbers of non-boxer athletes; the overwhelming majority from autopsy studies in former American footballers [28, 31], in addition to isolated descriptions of former athletes from a wide and growing range of sports [1, 9, 10, 22, 25, 37, 47], former military personnel [8, 38] and individuals exposed to a single moderate or severe TBI [15, 51]. Notably, many of these contemporary studies often describe more limited and localized pathology than historical case series of DP, with perhaps more attention on neuropsychiatric than cognitive symptomatology [42, 46]. Further, recent reporting largely focuses on tau pathologies in CTE over the constellation of non-tau proteinopathies that develop in late survivors from TBI. Reflecting this, preliminary consensus criteria for the neuropathological identification of CTE define the disease solely on the pattern and distribution of tau [26]. Finally, many reports often do not consider the clinical consequences of CTE pathology, particularly in patients with dementia where multiple neurodegenerative pathologies may co-exist.

There is, therefore, a continuing need to understand the complex neuropathology of late survival from exposure to TBI and its interaction with wider neurodegenerative pathologies. Herein, we report experience from a single institution on the neuropathological assessment of the largest series to date of former soccer (Association football) and rugby union (hereafter ‘rugby’) players with histories of dementia. Specifically, applying established and preliminary consensus criteria for neuropathological assessment of a range of neurodegenerative diseases, together with detailed review of the clinical histories, we provide insight into the integrated clinicopathological diagnoses of dementia in a cohort of former athletes exposed to repetitive head impacts and mild TBI. Our observations suggest that CTE neuropathologic change (CTE-NC) is present in a high proportion of former soccer and rugby players with dementia. However, considered together, the clinical features and neuropathologies support an integrated clinicopathologic diagnosis of CTE dementia (CTE-D) in only a small proportion of these cases.

## Methods

### Ethical approval and recruitment

Ethical approval for all procedures in this study was provided by: the University of Glasgow Medical Veterinary and Life Sciences College Ethics Committee (reference number 200160147); the West of Scotland Research Ethics Committee (project ID 225271); and the Greater Glasgow and Clyde Biorepository (Application number 340). In each case, written authorization for a hospital diagnostic autopsy and donation of brain tissue to the Glasgow TBI Archive for use in research was obtained from the next of kin. Nevertheless, to preserve patient confidentiality, specific details of individual cases are presented as summarized data, with age at disease onset and death presented in decades. Consecutive research brain donations spanning the period 2014–2018 were included: one case each from years 2014, 2015 and 2016; two from 2017; and six from 2018. No potential donors notified to the Archive over the period of accrual were excluded.

### Neuropathological evaluation

At the time of autopsy, whole brains were immersion fixed in 10% formal saline for a minimum of 2 weeks, following which intact brains, where available, were transported to Glasgow for dissection and processing. For Case 1, only tissue blocks from target areas were available for review. Case 7 had been partially dissected prior to transport, but the complete specimen remained available for assessment. Following fixation, all brains were dissected, examined macroscopically and processed to paraffin using standard laboratory techniques. Tissue blocks for histopathological evaluation were sampled in line with standardized protocols for assessment of neurodegenerative disease. Specifically, sampled tissue blocks were sufficient to fulfill the minimum sampling recommended by the preliminary consensus criteria for CTE [26].

From all tissue blocks, 8 μm sections were prepared and stained for Hematoxylin and eosin using standard laboratory techniques. In addition, in line with consensus protocols, sections from selected blocks were stained for: hyperphosphorylated tau (PHF-1; 1:1000; Dr P Davis), Aβ (6f3d; 1:75; Dako), phosphorylated TDP-43 (1D3; 1:500; Millipore), alpha-synuclein (KM51; 1:200; Leica) and, where indicated, 3-repeat (8E6/C11; 1:3000; Millipore) and 4-repeat tau (1E1/A6; 1:400; Millipore). Thereafter, stained sections were independently reviewed by two neuropathologists (KK, WS), blind to clinical history, and a consensus reached on the nature, extent and distribution of pathologies using established criteria for neuropathological assessment of neurodegenerative disease. Specifically, preliminary NINDS consensus diagnostic criteria were employed for assessment of CTE pathology [26], with CTE neuropathologic change (CTE-NC) confirmed where hyperphosphorylated tau was present in neurons, astrocytes and cell processes around small vessels in an irregular pattern and at the depths of cortical sulci. Further, although consensus staging criteria for CTE do not exist, where present, extent and distribution of CTE-NC were dichotomized as ‘low’ or ‘high’, corresponding to stages I/II or III/IV, respectively, of a widely used protocol [28].

### Verbal autopsy

Following completion of the neuropathology evaluation, face-to-face or telephone discussions (where face-to-face meeting was not practical) between the lead investigator (WS) and the next of kin were arranged. These comprised open ended, semi-structured interviews documenting a narrative account of the patient’s clinical course, dementia diagnosis in life and management in the final illness. In addition, details were gathered on lifelong health (including exposure to TBI), history of participation in sport (including combat sports), medications, drug and alcohol exposure, family history and history of employment. At the culmination of this verbal autopsy, the comprehensive clinical information obtained was considered in context of the neuropathological features and a layered, diagnostic autopsy opinion provided, encompassing the final integrated diagnosis of dementia (clinicopathological diagnosis of dementia subtype) [21].

### Statistics

Summary statistics and between-group differences were calculated using Minitab (Version 18.1, Minitab Inc.), with between-group differences assessed using the two-sample *t*-, Mann–Whitney or Kruskal–Wallis tests as appropriate.

## Results

### Demographic and background information

Eleven brain donations from former athletes with a history of dementia were received over the period of study (seven soccer; four rugby). Average age at onset of dementia was 60 ± 8.6 years (range 48–75), with no difference in age of onset between former soccer (59.7 ± 8.1 years) or rugby (60.5 ± 10.8 years; *p* = 0.906) players, and similar disease durations (soccer 9.4 ± 4.5 years; rugby 11.2 ± 6.8 years; *p* = 0.657). Mixed playing positions were reported, with years of active participation in their sport estimated at 23.8 ± 8.4 years (range 11–35 years; rugby 28.8 ± 6.5 years vs soccer 21 ± 8.5 years; *p* = 0.156). In five subjects, a history of previous TBI with brief loss of consciousness was reported; none required hospital admission and all would be compatible with ‘mild’ TBI/concussion. Full demographic information, including details of sports career and post-mortem intervals, is provided in Table 1.

**Table 1:** Cohort demographic information

Case no	Sex	Sport	Position	Other contact sports	Estimated years in sport	History of TBI with LOC	Family history NDD	PMI (days)
1	M	Soccer	Forward	No	18	NA	Nil	NA
2	M	Soccer	Defense	No	20	Yes	Nil	11
3	M	Soccer	Defense	No	35	Yes	Nil	4
4	M	Soccer	Defense	No	30	No	Mother and brother ‘dementia’	3
5	M	Soccer	Forward	No	18	No	Nil	1
6	M	Soccer	Defense	Rugby	15	No	Nil	3
7	M	Soccer	Defense	No	11	No	Mother PD	3
8	M	Rugby	Back	No	20	Yes	Nil	0.5
9	M	Rugby	Forward	No	35	Yes	Brother AD	2
10	M	Rugby	Forward	No	32	Yes	Nil	2
11	M	Rugby	Forward	Soccer	28	No	Nil	6

*AD* Alzheimer’s disease, *LOC* loss of consciousness for less than 30 min, *NDD* neurodegenerative disease, *PD* Parkinson’s disease, *PMI* post-mortem interval, *TBI* traumatic brain injury

## Neuropathology

### Macroscopic appearances

**Fig. 1:**
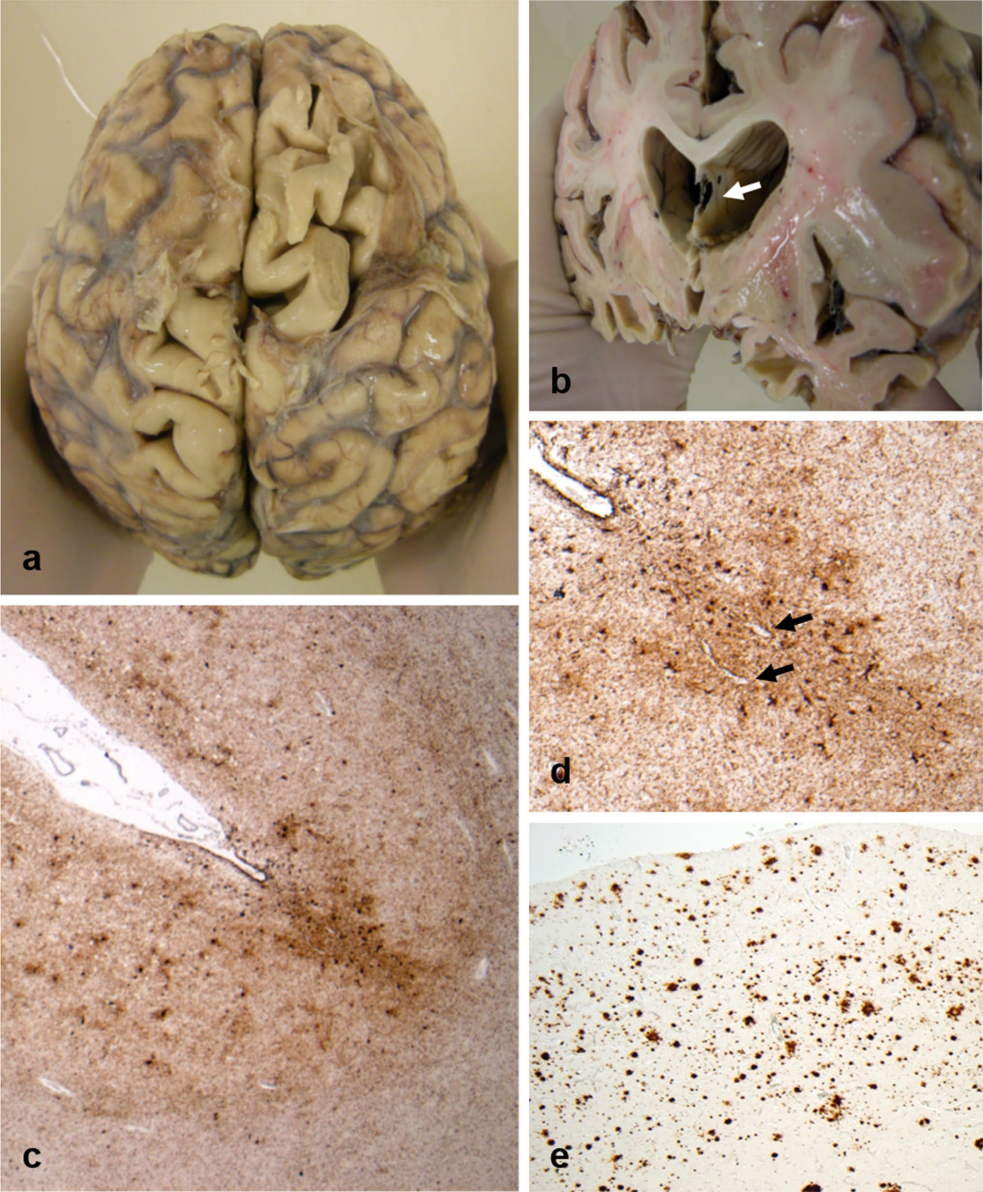
Representative macroscopic and histologic images from Case 8, a former rugby union player who first presented in his 50 s with a change in behavior marked by increasing levels of frustration and agitation associated with aggressive outbursts. In the following years, these behavioral and personality symptoms progressed, with cognitive disturbance evolving late in the course of disease. The patient died in his 70 s, 18 years after first presentation. On examination of the whole brain, there is gyral atrophy with associated sulcal thinning, most evident in frontal lobes (**a**). Sectioning the brain in the coronal plain through the mammillary bodies reveals ventriculomegally, consistent with the atrophy noted externally, in addition to fenestration of the septum pellucidum (**b**; white arrow). Staining sections for hyperphosphorylated tau (p-tau; PHF1) reveal widespread cortical immunoreactivity, within which are multiple patches of increased staining density towards the depths of cortical sulci (**c**), which on higher power are seen to coincide with neuronal and astroglial immunoreactive profiles clustered around small blood vessels (**d**; black arrows). In addition to widespread p-tau pathology, numerous Aβ plaques are present (**e**; 6f3d). The final integrated dementia diagnosis was CTE-D

Neuropathological evaluation revealed intact, fixed brain weights typically low (soccer 1225 ± 118 g; rugby 1224 ± 120 g; *p* = 0.989), with associated atrophy of frontal and temporal lobes (Fig. 1a) and hydrocephalus *ex vacuo*. In addition, moderate atrophy of the heads of the caudate nuclei was noted in Case 11. Septum pellucidum abnormalities were present in five cases, as fenestrated (Fig. 1b) or cavum septum or as mixed fenestrated and cavum (Table 2). Evidence of old, focal ischemic pathology as cavitated infarcts was noted in four cases. However, no focal pathology consistent with previous traumatic brain injury was present; specifically, no healed contusions were identified.

**Table 2:** Neuropathological observations

Case no	Sport	Septum	CTE-NC	ADNC			CAA	TDP-43	Other ND pathology	Clinical diagnosis	Integrated CPC diagnosis
				*A*	*B*	*C*					
1	Soccer	F + C	High	1	2	0	4	–	CVD	AD	CTE
2	Soccer	C	High	0	2	0	0	5	CVD; PART	AD	VaD
3	Soccer	F	High	2	3	3	2.25	–	DLB	AD	DLB
4	Soccer	NA	High	3	3	2	0	4	CVD; ARTAG	Mixed AD/VaD	CTE
5	Soccer	C	Low	3	3	2	3.5	3	CVD; DLB; ARTAG	Mixed AD/VaD	AD
6	Soccer	Intact	No	3	3	3	4	–	ARTAG	‘early onset dementia’	AD
7	Soccer	Intact	No	0	0	0	0	4	PSP	AD	PSP
8	Rugby	F	High	3	3	2	3	5	ARTAG	VaD	CTE
9	Rugby	F	Low	3	3	3	4	4	CVD; ARTAG	AD	AD
10	Rugby	Intact	Low	2	3	3	0.25	3	CVD	AD	Mixed AD/VaD
11	Rugby	Intact	No	0	0	0	0	–	CBD	CTE	CBD

*AD* Alzheimer’s disease, *ADNC* Alzheimer’s disease neuropathologic changes, *ARTAG* aging-related tau astrogliopathy, *C* cavum, *CAA* cerebral amyloid angiopathy [35]; *CBD* corticobasal degeneration,*CPC* clinicopathological correlation, *CTE-NC* chronic traumatic encephalopathy neuropathologic change, *CVD* chronic cerebrovascular disease, *DLB* dementia with Lewy bodies, *F* fenestrated, *NA* not assessed, *ND* neurodegenerative disease, *PART* primary age-related tauopathy, *PSP* progressive supranuclear palsy, *TDP-43* abnormally phosphorylated TDP-43 pathology [16], *VaD* vascular dementia

### Microscopic appearances

Histological examination revealed a variety of neuropathologies, including those fulfilling criteria for multiple neurodegenerative pathologies (Table 2). Evidence of old ischemic injuries was confirmed in Cases 2, 4, 5, 9 and 10 as variably sized foci of architectural disturbance with neuronal loss and astrocytic gliosis, occasionally with cystic degeneration and an established glial limitans. Bilateral hippocampal sclerosis was also present in Case 2. Marked substantia nigra pigment incontinence, neuronal loss and astrocytic gliosis were noted in Cases 3, 7 and 11, and occasional remaining neurons containing either classical Lewy bodies (Case 3) or globose tangles (Cases 7 and 11).

### Hyperphosphorylated tau

**Fig. 2:**
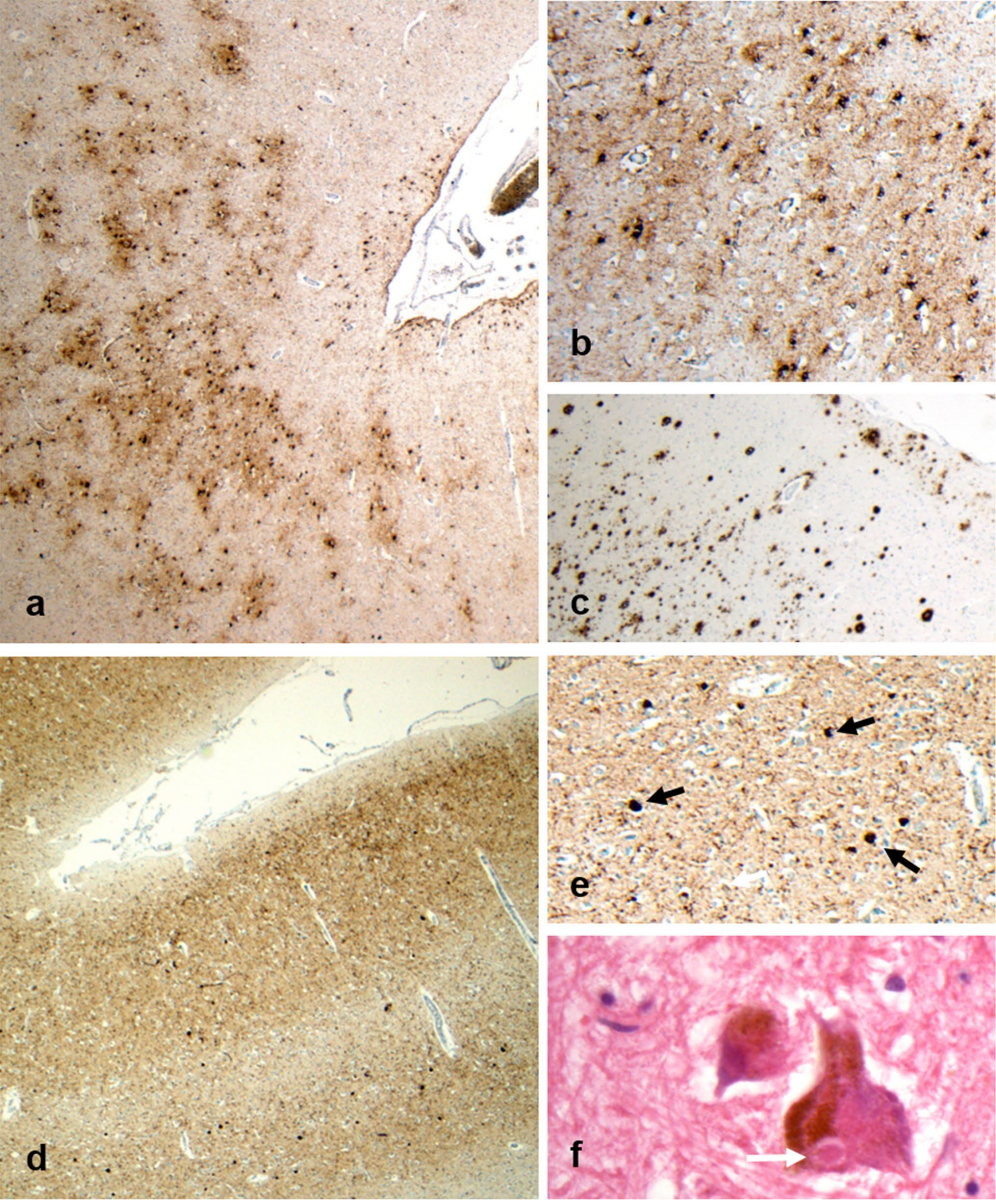
Representative images from Case 3, a former soccer player who first came to attention in his 60 s with mild cognitive impairment. There followed a steady progression in these symptoms, with exacerbations in the post-operative period, and with the development of visual hallucinations and motor signs leading to a clinical suspicion of Parkinson’s disease, or related disorder. After an 8-year course, the patient died in his 70 s. Staining for p-tau reveals patches of neuronal and astroglial immunoreactivity at the depths of cortical sulci (**a**), which on higher power are clustered around small cortical vessels (**b;** PHF1). Frequent Aβ plaques are also present (**c**; 6f3d). In addition, staining for alpha-synuclein reveals extensive cortical staining (**d**), with numerous cortical Lewy bodies present (**e**; black arrows). Sections of the midbrain also reveal scattered classical Lewy bodies (**f**; white arrow) within remaining pigmented neurons of the substantia nigra. Given the stereotypical clinical presentation and associated neuropathological features, an integrated diagnosis of dementia with Lewy bodies was made, with co-morbid CTE-NC noted

All 11 brains showed abnormal hyperphosphorylated tau (p-tau) pathology, consistent with several distinct tauopathies [17]. In eight cases (five soccer, three rugby), p-tau was present in a patchy distribution towards the depths of cortical sulci as neurofibrillary tangles, neurites and thorn-shaped astrocytes clustered around cortical vessels, fulfilling the preliminary consensus neuropathological criteria for the recognition of CTE (Figs. 1c, d, 2a, b) [26]. Notably, patients in whom CTE neuropathological change (CTE-NC) was present were generally older at disease onset (median 63.5 years vs 50 years; *p* = 0.019; Mann–Whitney) and at death (74.5 years vs 59 years; *p* = 0.041) than patients without CTE-NC. There was no difference in disease duration between cases with or without CTE-NC (10.5 years vs 8.7 years; *p* = 0.610).

In addition to CTE-NC, neurofibrillary tangle pathology in wider distribution was present in six former soccer and three former rugby players, consistent with AD Braak stage III to VI pathology [2]. In the remaining two cases, p-tau pathologies appeared as 4R tau-positive, 3R tau-negative, neuronal cytoplasmic inclusions, neurites, coiled bodies, and tufted astrocytes, or as astrocytic plaques, ballooned cells, and pre-tangles, consistent with pathologic diagnoses of progressive supranuclear palsy (PSP) [23] or corticobasal degeneration (CBD), respectively [5]. Finally, subcortical, periventricular, subpial or peri-infarct p-tau immunoreactive, and thorn-shaped astrocytes were present in five cases, typical of aging-related tau astrogliopathy (ARTAG) [19, 20].

#### Amyloid-β

Screening for amyloid-β (Aβ) pathologies revealed abnormalities in eight cases (five soccer, three rugby). In seven examples, these appeared as moderate to frequent, mixed diffuse and neuritic plaques (CERAD B or C) [32], typically in wide distribution (Thal phase 4 or 5) (Figs. 1e, 2c) [49], while in one case more localized (Thal phase 2) diffuse plaques were identified. In addition, cerebral amyloid angiopathy (CAA) was present in seven Aβ plaque-positive cases, typically of high severity by routinely employed histological assessment protocols [35].

#### TDP-43

Staining for phosphorylated TDP-43 revealed scattered neuronal cytoplasmic inclusions and neurites in seven brains, consistent with stage 3, 4 or 5 TDP-43 pathology [16]. This included Case 2, in which there was noted bilateral hippocampal sclerosis, supporting a pathological diagnosis of hippocampal sclerosis of aging [34]. The remaining cases were negative for TDP-43.

#### Alpha-synuclein

Alpha-synuclein-positive profiles were present in just two brains. In Case 3, abundant synuclein-immunoreactive neurites and neuronal cytoplasmic inclusions consistent with cortical Lewy bodies were present, supporting a neuropathologic diagnosis of diffuse neocortical pattern of Lewy body disease (Fig. 2d–f) [30, 39]. Case 5 exhibited classical Lewy bodies, with sparse synuclein-positive neurites and neurons in the cingulate gyrus, medial temporal lobe and amygdala supporting a diagnosis of limbic/transitional pattern Lewy body disease [30].

## Summary of neuropathologies

Thus, multiple, mixed neurodegenerative pathologies were typical in these former soccer and rugby players with dementia. In some form, p-tau pathologies were identified in all cases. Thereafter, Aβ pathologies were present in 73%, TDP-43 in 64%, vascular pathology in 55% and alpha-synuclein in 18%. Deposition of p-tau in a pattern and distribution fulfilling preliminary neuropathological criteria for CTE was present in eight cases, all but one of which also showed mixed p-tau and Aβ pathologies in keeping with Alzheimer’s disease neuropathologic changes (ADNC), with seven cases in total showing ‘high/intermediate’ ADNC and a single case with ‘low’ ADNC [33]. In addition to plaque pathology, widespread CAA was present in seven brains, five of which showed features of macro- and/or micro-vascular ischemic pathology. A further two cases had evidence of ischemic pathology in the absence of CAA. All five cases with abnormalities in the septum pellucidum had co-existent CTE neuropathology.

### Integrated clinicopathological diagnosis

Following neuropathological evaluation, verbal autopsy interviews with the donor’s next of kin, supplemented by clinical case records’ review, where available, provided comprehensive accounts of the clinical presentation and disease course (summarized in Table 3), which informed a final, integrated clinicopathological diagnosis of dementia subtype for each case. The most frequent integrated diagnosis was Alzheimer’s disease (AD; four cases; one as mixed AD and vascular dementia), followed by three cases with dementia compatible with CTE (CTE-D). Of the remaining cases, these included one case each of vascular dementia, dementia with Lewy bodies, PSP and CBD. Thus, within this cohort, three broad diagnostic categories might be proposed, distinguished by the presence of CTE-NC and its association with dementia diagnosis. These consist of: (1) patients with dementia but no evidence of CTE-NC (*n* = 3; median age onset 50 years); (2) patients with an integrated diagnosis of CTE-D (*n* = 3; median 55 years); and (3) patients with CTE-NC as co-morbidity in context of an alternate integrated diagnosis (*n* = 5; median 66 years; *p* = 0·024; Kruskal–Wallis).

**Table 3:** Major clinical symptoms reported

Case no	Age symptom onset	Age at death	Disease duration (years)	Major symptoms at presentation and over disease course
1	50 s	50 s	5	Behavioral change and personality change (impulsivity) at onset; progression to cognitive impairment (memory) late in disease
2	60 s	60 s	7	Mild, but evolving cognitive impairment (memory) at onset; acute stroke with cognitive deterioration later in disease and stepwise deterioration thereafter
3	60 s	70 s	8	Cognitive impairment (problem solving) at onset; acute deterioration post-operatively; Parkinsonism and visual hallucinations late in course
4	60 s	70 s	10	Behavioral change with aggression and cognitive impairment (memory) at onset; Reduced mobility late in disease
5	60 s	80 s	16	Loss of confidence associated with cognitive impairment (spatial awareness) at onset; progressing memory impairment; sleep and behavioral disturbance late in course
6	50 s	50 s	6	Cognitive impairment (memory; conceptual) at onset; behavioral change later as mild disinhibition
7	50 s	60 s	13	Behavioral change at onset; progressing to memory decline and motor symptoms
8	50 s	70 s	18	Behavioral and personality change (short tempered; irritable; loss of confidence); memory impairment late in disease
9	60 s	70 s	16	Cognitive impairment (memory) at onset; gradual decline with evolving cognitive symptoms thereafter; not behavioral symptoms
10	70 s	70 s	4	Cognitive impairment post diagnosed lacunar infarct at onset; decline thereafter
11	50 s	50 s	7	Behavioral change (becoming introverted) and mild cognitive (memory) symptoms at onset; motor symptoms (shuffling gait) and speech loss with progression

## Discussion

Herein, we present observations on neurodegenerative pathologies and their relationship to the final integrated, clinicopathological dementia diagnosis in a consecutive series of 11 brain donations from former soccer and rugby union players with dementia. In addition to ubiquitous tau pathologies, our cases invariably showed mixed, often multiple, co-existent neurodegenerative pathologies, including frequent Aβ pathologies as amyloid plaques and cerebral amyloid angiopathy. Further, although the pathognomonic neuropathology of CTE described within preliminary consensus criteria was present in a high proportion of brains in this series, in only three would CTE be regarded as the primary integrated dementia diagnosis following comprehensive review of the clinical histories and neuropathologies. In the remaining five cases with CTE neuropathologic change (CTE-NC), this appeared as co-morbid pathology in the setting of an alternative integrated dementia diagnosis. Thus, in context of dementia arising in athletes exposed to repetitive mild traumatic brain injury, three distinct clinicopathological groupings are suggested by this series defined by the presence of CTE-NC and its relationship to the final integrated dementia diagnosis. These observations support the need for comprehensive neuropathological evaluation and reporting in individuals exposed to TBI, with distinction between the autopsy finding of CTE-NC and the putative clinical consequences of this pathology, including CTE dementia (CTE-D).

Notably, mixed neurodegenerative pathologies were present in nine of our series, even in relatively younger aged patients. Mixed pathologies increasingly are recognized in studies of neurodegenerative disease [18, 41, 44], with prevalence estimates ranging up to 80% of dementias at autopsy and directly correlated with age [41]. In contrast to our experience, previous series report mixed neurodegenerative pathologies in just 45% of former athletes [31]. In this respect, mixed pathologies appear more frequent in our patients than in similar studies in former athletes, perhaps reflecting an older patient cohort. Conceivably, such mixed neurodegenerative pathologies might contribute to clinically ‘atypical’ presentations, leading to challenges in establishing accurate clinical diagnoses. There is undoubtedly a need for continued autopsy brain examination in individuals surviving TBI, with accurate and comprehensive documentation of the full spectrum of pathologies encountered in each examination.

Reporting in CTE largely focuses on p-tau pathologies. Indeed, preliminary consensus criteria define pathognomonic CTE neuropathology solely by the pattern and distribution of p-tau [26]. While pathognomonic p-tau pathologies were common in our current series, Aβ pathology as plaque and/or CAA was as frequent and present in all but one case with CTE-NC. In contrast, studies on former American footballers with CTE document Aβ pathology in just 61% [45]. However, incidence of dementia was reported higher in those older American footballers in which there were high burdens of CTE and Aβ. In this context, our data support these observations and might suggest that few post-TBI dementias arise in context of a pure tauopathy (pure CTE-NC), the majority arising in context of mixed proteinopathies, including frequent Aβ pathologies. As such, dementia-associated neurodegenerative pathology in former athletes might be more reminiscent of AD.

Since the first description of CTE in a former American football player [36], there has been a proliferation in autopsy series reporting its presence in individuals surviving a spectrum of TBI exposures (for review, see Hay et al. [11]). Nevertheless, the published case experience in CTE remains low [43], with reports typically documenting CTE pathology, but including little interpretation of its clinical implications. By example, an autopsy series documenting 110 of 111 former National Football League (NFL) American footballers with CTE pathology offered no insights into final integrated clinicopathological diagnoses [31]. Similarly, no opinion on final, integrated clinicopathological diagnoses after neuropathological evaluation was provided in a study of six former soccer players with dementia, four of whom had CTE neuropathology [22]. We identified CTE-NC in eight of our 11 former athletes with dementia. However, in just three of these cases was the integrated, primary dementia diagnosis consistent with CTE dementia (CTE-D) after consideration of the comprehensive clinical information. As such, while CTE-NC is a common pathology in former contact sports athletes with dementia, CTE-D appears a less common primary dementia diagnosis.

In this series of former soccer and rugby players with dementia, CTE-NC was present in 73% of cases. Adding this experience to observations in previous, isolated case reports in rugby and soccer, where sought, autopsy confirmed CTE pathology is reported in 75% of former athletes from these sports: 13/18 (seven from this study) soccer [1, 6, 9, 10, 22, 25] and 5/6 (four from this study) rugby [25, 45]. Elsewhere, CTE neuropathology has been reported ranging from 50% (7/14) of former boxers [7] to 99% (110/111) of former NFL footballers examined [31]. Although such studies are subject to numerous biases and limitations, rendering them uninformative regarding true disease prevalence, the apparent variation in reported prevalence of CTE neuropathology between these autopsy series might suggest between sports’ differences in risk and/or variability in methodologies of specimen accrual and neuropathology assessment between laboratories.

In summary, our observations suggest that while CTE-NC may be a common pathology in former soccer and rugby players with dementia, CTE dementia appears a less common primary dementia diagnosis. Current criteria for AD distinguish neurodegenerative pathologies, defined as AD neuropathologic changes (ADNC) [13, 33], from the clinical syndrome of dementia of Alzheimer’s disease type [14]. Reflecting this approach, we propose that there should be a similar distinction between CTE neuropathologic change and the clinical consequences of this pathology, including CTE dementia. As experience grows, CTE-NC conceivably might echo experience in AD, where the neuropathologic change can represent incidental pathology without clinical effect, or the major pathology driving clinical dementia phenotypes, or a co-morbid pathology within mixed neurodegenerative disease. In other words, in the absence of clinical correlation, CTE neuropathologic change should perhaps be regarded as a pattern of pathology, rather than diagnostic of a specific disease.
